# Impaired patient-reported outcomes but preserved gait patterns 5–15 years after acetabular fracture compared with healthy controls

**DOI:** 10.3389/fbioe.2026.1727785

**Published:** 2026-01-15

**Authors:** Selma Fensel-Merz, Elke Warmerdam, Marcel Orth, Tim Pohlemann, Emmanouil Liodakis, Bergita Ganse

**Affiliations:** 1 Department of Trauma, Hand and Reconstructive Surgery, Departments and Institutes of Surgery, Saarland University, Homburg, Germany; 2 Werner Siemens-Endowed Chair for Innovative Implant Development (Fracture Healing), Departments and Institutes of Surgery, Saarland University, Homburg, Germany

**Keywords:** acetabulum, gait analysis, ground reaction force, pain, PROMIS, treadmill, fracture healing, rehabilitation

## Abstract

**Introduction:**

Little is known about the long-term subjective patient experience after acetabular fracture and its relationship with changes in gait patterns. Worse outcomes were hypothesized compared with healthy control participants.

**Methods:**

Patient-Reported Outcome Measure Information System (PROMIS) questionnaires and treadmill-based gait analyses were conducted. Twenty parameters derived from the ground-reaction force curve were analysed. One-way ANOVA, Mann‒Whitney U tests, and regression statistics were used to assess differences between patients and controls (26 participants) and correlations between PROMIS scores and gait parameters.

**Results:**

Twenty-six patients (19 men and 7 women, 52.09 ± 12.77 years) with previous acetabular fracture an average of 9.90 ± 2.97 years prior to the study were included, all with excellent or good quality of reduction. While significantly worse results were found in the fracture than in the control group for all tested PROMIS scores (Physical Health, p < 0.001; Mental Health, p < 0.001; Pain Interference, p = 0.011; Physical Function, p < 0.001), no changes were observed in the gait parameters. There was no correlation between the PROMIS scores and any of the gait parameters. Forty-six percent of patients reported occasional pain and twelve percent noted weather sensitivity.

**Discussion:**

Factors other than changes in gait pattern seem to cause worse PROMIS scores in patients after acetabular fracture.

## 1 Introduction

Acetabular fracture results in substantial socioeconomic costs and a reduced quality of life (Hinz et al., 2023). Independent of the fracture type, acetabular fracture is associated with secondary degeneration of the hip joint cartilage in 20%–40% of patients (Gänss et al., 2024). In a study of 816 patients, the overall 10-year hip joint survival rate was 86%, and the 20-year survival rate was 79% (Tannast et al., 2012). Injury to the femoral head and acetabular impaction are the strongest predictors of failure of the hip joint (Tannast et al., 2012). Additionally, poor reduction, associated injuries, fracture displacement of more than 20 mm, joint dislocation and late surgery are associated with a poor prognosis (Meena et al., 2013). Posttraumatic osteoarthrosis, necrosis of the femoral head, infections, heterotopic ossification, nerve deficits and thromboembolic events are the leading complications in the healing process after acetabular fracture, resulting in pain and restricted movement (Hinz et al., 2023; Gänsslen et al., 2024; Briffa et al., 2011; Magu et al., 2014). Concomitant injuries (Matta, 1996), delayed surgical treatment and delayed rehabilitation, as well as the patient’s age (Matta, 1996), the quality of surgical treatment (Matta, 1996) and the type of fracture (Judet et al., 1964; Matta et al., 1986; Pennal et al., 1980; Oh et al., 2006), are decisive for the postoperative functional outcome.

In addition to these functional outcomes, patients with pelvic and acetabular fractures are at increased risk for insomnia and sleep disorders (Ali et al., 2024). Their psychological wellbeing varies among fracture types, with signs of borderline clinical depression observed in some cases (Ali et al., 2024). However, little is currently known about the subjective patient experience after acetabular fracture. Among the tools used to study patient experience is the Patient-Reported Outcome Measure Information System (PROMIS), which comprises numerous questionnaires of different aspects of recovery (Rothrock et al., 2020). It was developed with National Institutes of Health (NIH) funding and is now among the most popular questionnaire systems used in orthopaedic surgery (Gilat et al., 2023). Indeed, the PROMIS was found to be a more efficient instrument for evaluating patients with acetabular fractures than longer instruments, such as the Short Musculoskeletal Function Assessment (SMFA) and the Short Form 36 (SF-36) questionnaires (Schumaier et al., 2022).

Among 63 teenagers with a mean follow-up time of 3.5 years after pelvic/acetabular fracture, more than 85% reported no or only mild concerns in several PROMIS questionnaires (Hailer et al., 2024). In the domain of pain interference, 11% indicated moderate concerns, and 2% indicated considerate concerns (Hailer et al., 2024). In the domain mobility, however, 12% indicated moderate concerns, and 3% indicated considerable concerns. Despite this finding in the mobility domain, details on gait or other mobility functions were not correlated with the PROMIS score in a study by Hailer et al. (2024) or in other previous studies. It is therefore of interest to study the associations of changes in gait patterns with PROMIS outcomes after acetabular fracture. Apart from adolescent patients, owing to the higher incidence, such studies seem to be of particular importance for adult patients and their longer-term outcomes.

Studies on long-term changes in gait patterns after acetabular fractures are rare. In 19 patients, most of the kinematic and kinetic variables fully recovered 12 months after surgery for acetabular fracture, whereas anterior pelvic tilt and hip abduction moment still differed (Kubota et al., 2012). Differences in kinematics, particularly larger knee flexion angles, reduced maximal ankle dorsiflexion, and less hip rotation (mainly with a posterior than an anterior surgical approach), were detected in 30 patients (Engsberg et al., 2009). Losses in muscle strength seem to be the major causes of these reported changes, particularly strength deficits in the hip abductors (Kubota et al., 2012; Engsberg et al., 2009). However, longer-term gait data after acetabular fracture have not yet been reported.

For these reasons, the aim of this study was to study long-term PROMIS score and gait outcomes in patients after acetabular fracture. It seems obvious that impaired gait would negatively affect physical and mental health scores, and possibly pain. However, additional variables other than gait likely affect the separate PROMIS outcomes. Therefore, the aim was to study how PROMIS scores and gait parameters correlate in this patient collective. The following null-hypothesis was proposed: Five to 15 years after acetabular fracture, in patients who did not undergo total hip arthroplasty (THA), 1. PROMIS scores and 2. Gait patterns are worse than those of matched healthy control participants. In addition, we hypothesized the following: 3. There are no significant correlations between any of the PROMIS scores and any of the gait parameters; and 4. these patients report problems in daily life related to the acetabular fracture.

## 2 Methods

This observational retrospective cohort study was approved by the ethics committee of the Saarland Medical Association, identification number 30/21. It was registered in the German clinical study register (DRKS00034640) prior to the start of patient recruitment. The cross-sectional study design included a single laboratory assessment of each patient and participant.

### 2.1 Participants

Data were collected on a voluntary basis from healthy volunteers recruited from staff and patients’ relatives, as well as from patients of both sexes who had been treated for an acetabular fracture at Saarland University Hospital five to 15 years previously. This lengthy time period was selected to be able to collect a large number of patients. The healthy volunteer group included people of both sexes between the ages of 18 and 75 years without preexisting injury to the pelvis or legs. The exclusion criteria were the requirement of walking aids that must not be used on a treadmill, THA, inability to provide informed consent, or pregnancy. Patients of both sexes between the ages of 18 and 75 years who had been treated for an acetabular fracture between 5 and 15 years prior were retrospectively contacted by telephone. Patients who had received either surgical or nonoperative treatment were first screened for the inclusion and exclusion criteria in an initial interview. The exclusion criteria were further injuries to the lower limbs, the requirement of walking aids that must not be used on a treadmill, inability to provide informed consent, or pregnancy.

### 2.2 Measurement protocol

To address the stated hypotheses, the single laboratory assessment included the completion of PROMIS questionnaires and a walking experiment on an instrumented treadmill. In addition, hand grip strength, body height and weight were measured for group matching.

The following PROMIS questionnaires (No authors listed. Health Measures - Northwestern University, 2024) were collected:

- PROMIS Global Health (v1.2)
- PROMIS Pain Interference (v1.0, Short Form 8a)
- PROMIS Physical Function (Short Form 14)

The PROMIS Global Health questionnaire comprises ten items and can be differentiated into mental and physical health domains (No authors listed. Health Measures - Northwestern University, 2024). The PROMIS Pain Interference questionnaire uses eight items to assess the extent to which pain influences social activities and participation. The PROMIS Physical Function questionnaire uses 14 categories to test general physical functioning. In addition to the PROMIS questionnaires, the patients were given the opportunity to write down problems they experienced in their daily life. This item was structured as a single, open-ended question.

**FIGURE 1:**
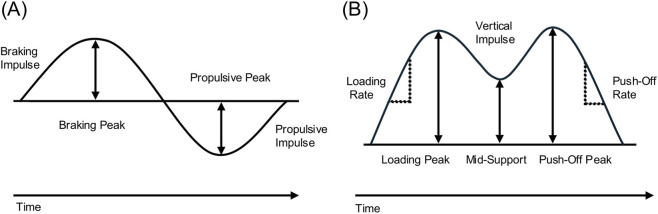
**(A)** Visualization of the gait parameters in the anterior-posterior and **(B)** vertical orientations. All of the analysed outcome parameters were based on the GRF curve during the stance phase. GRF data were recorded for 1 minute at a walking speed of 4 km/h. The measurement started as soon as a safe gait pattern could be recognized and the patients reported feeling that their gait was safe. No additional kinematic data were recorded.

For gait analysis, the participants and patients walked on a level treadmill instrumented with four pressure sensors (Gaitway 3D, hp cosmos, Nussdorf, Traunstein, Germany). The extracted outcome parameters are described in Figure 1 and Table 1.

**TABLE 1:** Descriptions of the gait parameters obtained in this study. On the basis of the raw GRF data, these gait parameters were computed by the software provided for the Gaitway 3D treadmill (Arsalis SRI version 1.7.10; Glabais, Belgium; and Hp cosmos Nussdorf, Traunstein, Germany).

Parameter	Description	Unit
Vertical impulse	Force over the entire stance phase	N·s
Loading rate	Increase in force during the loading phase	N·s
Loading peak	Time of first force maximum at the end of the loading phase	s
Time to loading peak	Time to loading peak	s
Mid-support force	Minimum force between the two maxima	N
Time to mid-support	Time to mid-support force	s
Push-off peak force	Second force maximum, defined as the start of the push-off phase	N
Time to push-off peak	Time to push-off peak	s
Push-off rate	Decrease in force during the push-off phase	N/s
Braking impulse	Force over the braking phase	N·s
Braking peak force	Maximum force during the braking phase	N
Time to braking peak	Time to braking peak	s
Propulsive impulse	Force over the propulsive phase	N/s
Propulsive peak force	Maximal force exerted during the propulsive phase	N
Time to propulsive peak	Time to the propulsive peak	s
Contact duration	Total contact time	s
Step duration	Duration of a step - from initial contact of one foot to initial contact of contralateral foot	s
Double support duration	Time that both feet are in contact with the ground during one step	s
Single support duration	Time that only one foot is in contact with the ground during a step	s
Stride duration	Time to complete a stride - from initial contact to initial contact of the ipsilateral foot	s

In addition, hand grip strength was recorded as a measure of overall muscle strength and fitness using a hand dynamometer (Kern MAP 130K1, Balingen, Germany). The measurement was taken in a seated position with the elbow bent at 90°. The participants were asked to perform three maximal contractions with the dominant hand. The average of the three values was used for analyses. Body weight was assessed using a regular digital bathroom scale (Beurer MS 50, Ulm, Germany). Body height was measured with a Seca 206 roll-up measuring tape with wall attachment (Seca, Hamburg, Germany). As grip strength, body height, body weight and age are known to affect ground-reaction force (GRF)-derived gait parameters (Wolff et al., 2023), the control group was matched on the basis of these factors.

### 2.3 Quality of reduction

To assess the quality of the fracture reduction according to Matta (1996), radiographs or computed tomography (CT) scans taken immediately after the operation and no later images were classified by an experienced orthopaedic surgeon for this analysis. Matta’s radiographic outcome grading comprises four categories, excellent, good, fair and poor. These categories were applied accordingly. The quality of reduction in terms of an anatomical reconstruction has significant relevance for the development of arthritis (Gänsslen et al., 2024; Meena et al., 2013).

### 2.4 Statistical analysis

The data were analysed using SPSS, version 30.0 (SPSS Inc., Chicago, USA). After the data were tested for a normal distribution with the Kolmogorov‒Smirnov and Shapiro‒Wilk tests, in the case of a normal distribution, one-way ANOVA was conducted with post hoc tests using Bonferroni correction. In the absence of a normal distribution, the Mann‒Whitney U test was applied. Linear regression statistics and one-way ANOVA were used to determine if the PROMIS score and gait parameters were correlated. Continuous variables are expressed as the means ± standard deviations. Nominal-scale variables are given as numbers (n). Significance was assumed at p < 0.05.

## 3 Results

Twenty-six patients (19 men and 7 women aged 52.09 ± 12.77 years) who had previously experienced acetabular fracture an average of 9.90 ± 2.97 years prior to the study were included (Table 2). Details on the patients included in this study are provided in Supplementary Table 1. The control group comprised 26 matched healthy participants (19 men and 7 women aged 52.99 ± 12.70 years). Among the 26 fractures, according to the classification by Judet and Letournel (Judet et al., 1964), 8 (31%) were of the anterior column combined with a posterior hemi-transverse fracture type; 6 (23%) were posterior wall fractures, 4 (15%) were fractures of both columns, 4 (15%) were anterior column fractures, 2 (8%) were t-shaped, one was a transverse column fracture (4%) and one was a posterior column fracture (4%). The quality of the reduction according to the classification by Matta (1996) was either excellent or good in all of these cases. None of the patients had a fair and poor reduction.

**TABLE 2:** Patient and participant characteristics.

​	Sex [m/f]	Age [years]	Height [m]	Weight [kg]	Grip strength [kg]
Patients	19/7	52.09 ± 12.77	1.77 ± 0.09	85.01 ± 18.46	38.55 ± 12.67
Healthy control participants	19/7	52.99 ± 12.70	1.77 ± 0.08	84.45 ± 15.38	36.63 ± 10.07
P value	​	0.803	0.906	0.775	0.549

### 3.1 Questionnaires

**FIGURE 2:**
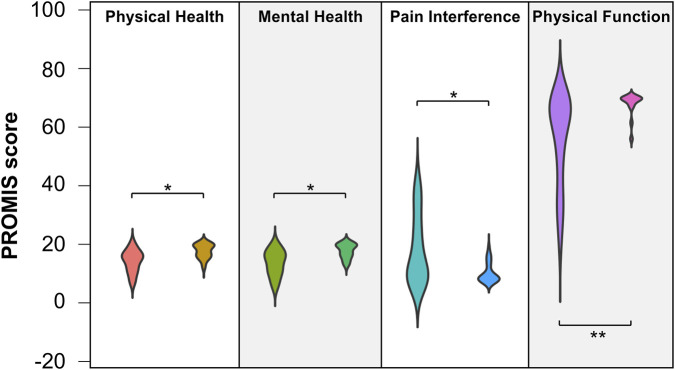
Violin plots of the PROMIS scores in the Global Physical and Mental Health, Pain Interference and Physical Function categories for patients (left) and healthy controls (right). The data are shown in absolute values. For Physical and Mental Health and for Physical Function, higher scores reflect a better outcome, whereas for Pain Interference, lower scores indicate a better result. Compared with the control group, the patient group scored significantly worse. The healthy test subjects reported little to no mental or physical impairment with respect to their general state of health. Compared with the patients surveyed, they also reported almost complete physical functioning and little to no impairment due to pain. * <0.05, ** <0.001.

The results of the PROMIS questionnaires are shown in Figure 2. Significantly worse PROMIS scores were reported by the acetabular fracture patients compared with the healthy control group in all tested PROMIS categories. In response to the additional question on problems in daily life, 12 patients (46%) answered that they had none. Twelve of the patients (46%) reported occasional pain either when driving the car, when lying in bed, when sitting or unrelated to what they did. Three of the patients (12%) noted weather sensitivity.

### 3.2 Gait analyses

**FIGURE 3:**
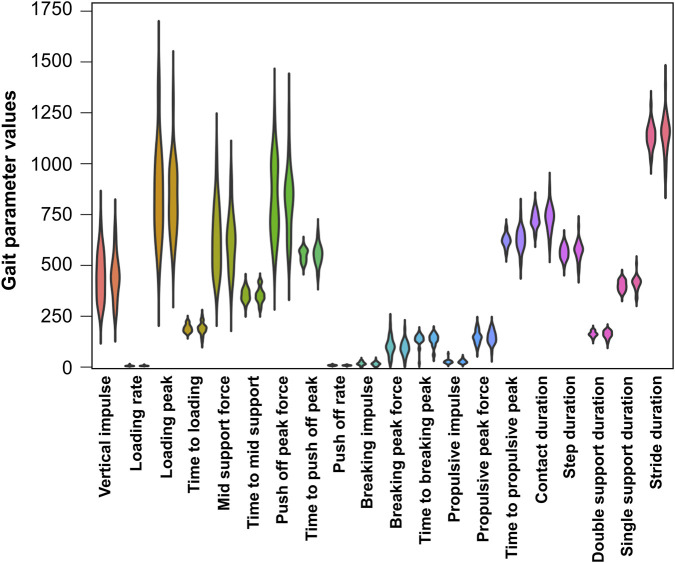
Violin plots showing the results of the gait analyses. For each gait parameter, the patient data are shown on the left, and the data of the healthy controls are shown on the right. There was no significant group difference in any of the parameters. The units are shown in Table 1.

The findings of the gait analyses are shown in Figure 3. No significant differences were found between the patients and the healthy control subjects in any of the tested gait parameters. The p-values are shown in Supplementary Table 2.

### 3.3 Correlation

There was no correlation between any of the PROMIS parameters and any of the gait parameters (Table 3). This was true for the different gait parameter types, the GRF-based parameters, the force and impulse parameters alike. High variation was found in the data, as indicated by the low R^2^ values shown in Table 3.

**TABLE 3:** Findings of the regression analyses. P values and *R* ^2^ values are shown separated by a semicolon.

Gait parameter	PROMIS global health, physical	PROMIS global health, mental	PROMIS pain interference	PROMIS physical function
Vertical impulse	0.294; 0.500	0.693; 0.007	0.096; 0.121	0.263; 0.057
Loading rate	0.538; 0.170	0.695; 0.007	0.830; 0.002	0.229; 0.065
Loading peak	0.509; 0.020	0.746; 0.005	0.216; 0.069	0.316; 0.046
Time to loading	0.114; 0.110	0.691; 0.013	0.105; 0.115	0.904; 0.001
Mid-support force	0.248; 0.060	0.583; 0.014	0.112; 0.111	0.218; 0.068
Time to mid-support	0.172; 0.830	0.581; 0.014	0.066; 0.146	0.739; 0.005
Push-off peak force	0.555; 0.016	0.793; 0.003	0.135; 0.099	0.406; 0.032
Time to push-off peak	0.342; 0.041	0.921; 0.000	0.139; 0.097	0.692; 0.007
Push-off rate	0.817; 0.002	0.839; 0.002	0.559; 0.016	0.717; 0.006
Braking impulse	0.795; 0.003	0.609; 0.012	0.696; 0.000	0.959; 0.000
Braking peak force	0.513; 0.020	0.429; 0.029	0.857; 0.002	0.857; 0.002
Time to braking peak	0.656; 0.009	0.931; 0.000	0.540; 0.017	0.251; 0.059
Propulsive impulse	0.461; 0.025	0.750; 0.005	0.073; 0.139	0.349; 0.040
Propulsive peak force	0.806; 0.003	0.646; 0.010	0.163; 0.087	0.531; 0.018
Time to propulsive peak	0.186; 0.078	0.930; 0.000	0.155; 0.090	0.715; 0.006
Contact duration	0.102; 0.117	0.703; 0.007	0.930; 0.123	0.502; 0.021
Step duration	0.151; 0.092	0.885; 0.001	0.192; 0.076	0.497; 0.021
Double support duration	0.138; 0.097	0.773; 0.004	0.033; 0.191	0.685; 0.008
Single support duration	0.282; 0.052	0.971; 0.000	0.552; 0.016	0.501; 0.021
Stride duration	0.086; 0.128	0.673; 0.008	0.072; 0.139	0.281; 0.053

## 4 Discussion

In this study, long-term changes 5–15 years after acetabular fracture were investigated using gait analyses and PROMIS questionnaires. While significantly worse results were found in the acetabular fracture group for all tested PROMIS scores, no changes were observed in the GRF-based gait pattern compared with matched healthy control subjects. In addition, no correlation was found between the separate PROMIS scores and any of the gait parameters.

Only patients who had not undergone THA were included in this study, and those who developed severe arthritis of the hip joint within the first few years were excluded. This is relevant, as patients with native hips reported overall superior outcome scores compared with THA patients following acetabular fracture fixation (Verbeek et al., 2018). However, the patients in this study still had extensive subjective limitations following the injury that were not reflected in the objective findings of the treadmill-based gait analyses. Thus, GRF-based gait analyses alone do not seem to be a suitable method for assessing long-term outcomes after acetabular fracture. Gait impairments were certainly not the primary cause of the decreased PROMIS scores, which could have been considered a possibility on the basis of a study conducted by Hailer et al. (2024), where mobility concerns were raised by acetabular fracture patients. In addition, significant reductions in the vertical ground reaction force and knee and hip extension moments have been reported 4 months after acetabular surgery for acetabular fractures treated using the pararectus approach (Brand et al., 2022).

In the present study, the relatively long time period of five to 15 years postoperatively was selected to collect enough patients for a meaningful analysis. Indeed, this is a long period that could by itself be associated with longitudinal and possibly even age-related changes. However, it is known that after pelvic and acetabular fracture, recovery of gait is usually already complete after 12 months, while pelvic forward tilt and hip abduction moment were the only parameters that still showed deviation after 12 months (Engsberg et al., 2009). Likewise, in fractures of the lower leg, gait and PROMIS parameters returned to normal in a 12-months period (Warmerdam et al., 2025). Therefore, in this study, it was assumed that no major changes in gait or PROMIS will occur within five to 15 years after surgery.

In the present study, all four PROMIS domains were impaired. However, these changes cannot be explained by changes in gait patterns. Thus, a possible explanation for the poor PROMIS outcomes is pain. In response to the question on problems in daily life, 46% of the patients after acetabular fracture reported occasional pain either when driving their car, lying in bed, or sitting or regardless of what they did. In addition, 12% of the patients noted weather sensitivity. Pain and functional impairment are known to be correlated (MacCormick and Sharma, 2018). According to the results of this study, pain does not affect gait patterns; therefore, experienced pain is likely not limited to characteristic osteoarthritic pain of the hip. Characteristic gait changes associated with hip osteoarthritis pain include an increase in trunk forward flexion, decreased stride length, increased hip extension and hip abduction during walking, and reductions in vertical GRF and knee and hip extension moments (Davis-Wilson et al., 2024; Brand et al., 2022; Chiodo, 2007; Muzii et al., 2021). These changes are considered caused by decreases in muscle strength (Kubota et al., 2012) and may be improved by maximizing hip muscle strength (Engsberg et al., 2009). These characteristic changes would be expected to affect the GRF curve of the stance phase due to less vigorous and more careful gait, including increases in loading rate and stride duration, as well as decreased peak forces. However, this study did not show any GRF-based gait changes compared with healthy control participants. Thus, worse PROMIS outcomes are associated with causes other than pain due to hip osteoarthritis. Pain properties and intensities have been reported to change over time following trauma (Chiodo, 2007). Pain appears to evolve from initial fracture pain and associated neuropathic pain to pain at the surgical site, ultimately progressing to pain caused by osteoarthritis. In 2007, Chiodo. (2007) investigated pain and gait alterations in patients with acetabular fractures, accounting for radiological results and electrodiagnostic measurements over 1 year postoperatively. Neuropathic pain was correlated with the extent of nerve damage but was not correlated with the extent of gait disturbance. In addition, studies have shown that neuroplasticity may cause neuropathic pain in response to nerve damage, which may occur adjacent to or remotely from the site of injury (Hiraga et al., 2022). Osteoarthritis is a disease with systemic implications, such as metabolic dysfunction and low-grade infection, obesity, sarcopenia and fragility, that may additionally affect pain perception (Herrero-Beaumont et al., 2024). This is particularly relevant, as acetabular injury influences the patient’s mental health, as indicated by reductions in the SF-12 score (Brand et al., 2022). Thus, future studies should aim to uncover the details of pain and possible treatment options after surgery for acetabular fracture.

Apart from pain, other factors could have affected the PROMIS score but were not reported because the participants were not aware of them or felt ashamed of them. Among those factors could be erectile dysfunction following injury- and surgery-related nerve injury (Elliott et al., 2023). In this study, none of the patients actively reported such problems, but an increased rate of erectile dysfunction was detected in patients after acetabular fracture at medium-term follow-up (Elliott et al., 2023). The quality of life was shown to be significantly decreased in female and male patients after pelvic fracture, with sexual dysfunction shown to be an independent risk factor for decreased quality of life after injury (Harvey-Kelly et al., 2014). Therefore, these issues should be separately and explicitly assessed in future studies (Machtens et al., 2001; Pohlemann et al., 1998).

Clinical implications of the present findings mainly include consequences for expectation management. This means that patients should be informed on the fact that possible longer-term negative effects of their physical and mental health, pain and physical function are to be expected. In case of such impairments, patients should not hesitate to seek professional help. Likewise, patients should be informed on the finding that gait will likely not be affected long-term.

This study had several limitations. The gait analyses were solely based on GRF-based parameters, whereas analyses of kinetics and kinematics, including joint angles, may provide further insight. In addition, gait is usually altered when a person is walking on a treadmill compared with an overground gait (Strutzenberger et al., 2022). Moreover, treadmill walking only involves straight walking; therefore, the gait assessed in this study might not be representative of daily living gait or mobility. Furthermore, the questionnaires were limited to several PROMIS questionnaires and one additional open question. In future studies, more specific questions on expected or frequent problems, as well as the types of pain and mental disorders, including questionnaires for depression, may be of interest. Structured interviews may be an option to improve the methodology. In future studies with larger patient groups, the associations of the PROMIS findings and gait parameters with the radiographic degree of arthritis and the quality of postoperative fracture reduction may be analysed. Subgroup analyses of individual acetabular fracture types may be possible in studies with larger sample sizes. Likewise, the separate surgical interventions could be individually correlated with the PROMIS outcomes. This analysis was unfortunately impossible in the present study due to small group sizes. In addition, future studies may be planned with a longitudinal design to identify the trajectories of the recovery-parameters over time.

## 5 Conclusion

Patients 5–15 years after acetabular fracture had worse PROMIS scores than did the matched healthy control participants. Despite these findings, neither differences were found in the analysed gait patterns of the same patients, nor was there any correlation between one of the PROMIS scores and any of the gait parameters. Thus, factors other than changes in gait seem to cause decreased PROMIS scores in patients after acetabular fracture. Forty-six percent of the patients reported occasional pain either when driving the car, when lying in bed, when sitting or unrelated to what they did. Twelve percent of the patients noted weather sensitivity. Further causes for worse PROMIS outcomes may include pain unrelated to hip osteoarthritis, such as neuropathic pain, or problems related to sexual function. Future studies are needed to investigate the causes of the decreases in PROMIS scores in this population and develop possible interventions.

## Data Availability

The original contributions presented in the study are included in the article/Supplementary Material, further inquiries can be directed to the corresponding author.

## References

[B1] Ali K. A. He L. Li W. Zhang W. Huang H. (2024). Sleep quality and psychological health in patients with pelvic and acetabulum fractures: a cross-sectional study. BMC Geriatr. 24 (1), 314. 10.1186/s12877-024-04929-y 38575871 PMC10993547

[B2] Brand A. von Rüden C. Probst C. Wenzel L. Augat P. Perl M. (2022). Early biomechanical outcome in patients with acetabular fractures treated using the pararectus approach: a gait and stair climb analysis study. Eur. J. Trauma Emerg. Surg. 48 (2), 1307–1316. 10.1007/s00068-021-01655-7 33835187 PMC9001237

[B3] Briffa N. Pearce R. Hill A. M. Bircher M. (2011). Outcomes of acetabular fracture fixation with ten years' follow-up. J. Bone Jt. Surg. Br. 93 (2), 229–236. 10.1302/0301-620X.93B2.24056 21282764

[B4] Chiodo A. (2007). Neurologic injury associated with pelvic trauma: radiology and electrodiagnosis evaluation and their relationships to pain and gait outcome. Arch. Phys. Med. Rehabil. 88 (9), 1171–1176. 10.1016/j.apmr.2007.06.004 17826464

[B5] Davis-Wilson H. Hoffman R. Cheuy V. Christensen J. Forster J. E. Judd D. L. (2024). Gait compensations, pain, and functional performance during the six minute walk test in individuals with unilateral hip osteoarthritis. Clin. Biomech. (Bristol) 120, 106366. 10.1016/j.clinbiomech.2024.106366 39490051 PMC11789618

[B6] Elliott I. S. Kleweno C. Agel J. Coale M. Patterson J. T. Firoozabadi R. (2023). Erectile dysfunction after acetabular fracture. OTA Int. 6 (2), e276. 10.1097/OI9.0000000000000276 37214108 PMC10194699

[B7] Engsberg J. R. Steger-May K. Anglen J. O. Borrelli J. (2009). An analysis of gait changes and functional outcome in patients surgically treated for displaced acetabular fractures. J. Orthop. Trauma 23 (5), 346–353. 10.1097/BOT.0b013e3181a278cc 19390362

[B8] Gänsslen A. Lindahl J. Staresinic M. Krappinger D. (2024). Outcomes of acetabular fractures. Arch. Orthop. Trauma Surg. 144 (10), 4641–4654. 10.1007/s00402-024-05596-9 39349875

[B9] Gilat R. Mitchnik I. Y. Patel S. Dubin J. A. Agar G. Tamir E. (2023). Pearls and pitfalls of PROMIS clinically significant outcomes in orthopaedic surgery. Arch. Orthop. Trauma Surg. 143 (11), 6617–6629. 10.1007/s00402-023-04983-y 37436494

[B10] Hailer Y. D. Larsson L. A. Hellström T. Chaplin J. E. Wolf O. (2024). Epidemiology and patient-reported measurement outcome of pelvic fractures in children and adolescents - a population-based cohort study from the Swedish fracture register. Injury 55 (8), 111700. 10.1016/j.injury.2024.111700 38941910

[B11] Harvey-Kelly K. F. Kanakaris N. K. Obakponovwe O. West R. M. Giannoudis P. V. (2014). Quality of life and sexual function after traumatic pelvic fracture. J. Orthop. Trauma 28 (1), 28–35. 10.1097/BOT.0b013e31828fc063 23481925

[B12] Herrero-Beaumont G. Castro-Dominguez F. Migliore A. Naredo E. Largo R. Reginster J. Y. (2024). Systemic osteoarthritis: the difficulty of categorically naming a continuous condition. Aging Clin. Exp. Res. 36 (1), 45. 10.1007/s40520-024-02714-w 38376694 PMC10879223

[B13] Hinz N. Dehoust J. Seide K. Kowald B. Mangelsdorf S. Frosch K. H. (2023). Epidemiology and socioeconomic consequences of work-related pelvic and acetabular fractures recorded in the German social accident insurance. Injury 54 (8), 110848. 10.1016/j.injury.2023.110848 37258403

[B14] Hiraga S. I. Itokazu T. Nishibe M. Yamashita T. (2022). Neuroplasticity related to chronic pain and its modulation by microglia. Inflamm. Regen. 42 (1), 15. 10.1186/s41232-022-00199-6 35501933 PMC9063368

[B15] Judet R. Judet J. Letournel E. (1964). Fractures of the acetabulum: classification and surgical approaches for open reduction: preliminary report. JBJS 46 (8), 1615–1675. 10.2106/00004623-196446080-00001 14239854

[B16] Kubota M. Uchida K. Kokubo Y. Shimada S. Matsuo H. Yayama T. (2012). Changes in gait pattern and hip muscle strength after open reduction and internal fixation of acetabular fracture. Arch. Phys. Med. Rehabil. 93 (11), 2015–2021. 10.1016/j.apmr.2012.01.016 22475054

[B17] MacCormick A. P. Sharma H. (2018). Does the severity of pain correlate with severity of functional disability? Factors influencing ‘patient reported outcome measures’ in spinal patients. SICOT-J. 4, 43. 10.1051/sicotj/2018029 30270822 PMC6166414

[B18] Machtens S. Gänsslen A. Pohlemann T. Stief C. G. (2001). Erectile dysfunction in relation to traumatic pelvic injuries or pelvic fractures. BJU Int. 87 (5), 441–448. 10.1046/j.1464-410x.2001.02147.x 11298032

[B19] Magu N. K. Gogna P. Singh A. Singla R. Rohilla R. Batra A. (2014). Long term results after surgical management of posterior wall acetabular fractures. J. Orthop. Traumatol. 15 (3), 173–179. 10.1007/s10195-014-0297-8 24879360 PMC4182623

[B20] Matta J. M. (1996). Fractures of the acetabulum: accuracy of reduction and clinical results in patients managed operatively within three weeks after the injury. J. Bone Jt. Surg. Am. 78 (11), 1632–1645. 10.2106/00004623-199611000-00002 8934477

[B21] Matta J. M. Mehnke D. K. Rom R. (1986). Fractures of the acetabulum: early results of a prospective study. Clin. Orthop. Relat. Res. 205, 241–250. 10.1097/00003086-198604000-00030 3698383

[B22] Meena U. K. Tripathy S. K. Sen R. K. Aggarwal S. Behera P. (2013). Predictors of postoperative outcome for acetabular fractures. Orthop. Traumatol. Surg. Res. 99 (8), 929–935. 10.1016/j.otsr.2013.09.004 24183746

[B23] Muzii V. F. Rollo G. Rocca G. Erasmo R. Falzarano G. Liuzza F. (2021). Radiographic and functional outcome of complex acetabular fractures: implications of open reduction in spinopelvic balance, gait and quality of life. Med. Glas. (Zenica) 18 (1), 273–279. 10.17392/1300-21 33219639

[B24] No authors listed. Health Measures - Northwestern University (2024). PROMIS. Obtain & Adm. Meas. Available online at: https://www.healthmeasures.net/explore-measurement-systems/promis/obtain-administer-measures (date last accessed November 28, 2024).

[B25] Oh C.-W. Kim P.-T. Park B.-C. Kim S.-Y. Kyung H.-S. Jeon I.-H. (2006). Results after operative treatment of transverse acetabular fractures. J. Orthop. Sci. 11 (5), 478–484. 10.1007/s00776-006-1045-6 17013736

[B26] Pennal G. F. Davidson J. Garside H. Plewes J. (1980). Results of treatment of acetabular fractures. Clin. Orthop. Relat. Res. 151 (151), 115–123. 10.1097/00003086-198009000-00014 7418294

[B27] Pohlemann T. Gänsslen A. Stief C. H. (1998). Komplexe Verletzungen des Beckens und Actetabulums. Orthopade 27 (1), 32–44. 10.1007/s001320050200 9540100

[B28] Rothrock N. E. Amtmann D. Cook K. F. (2020). Development and validation of an interpretive guide for PROMIS scores. J. Patient Rep. Outcomes 4 (1), 16. 10.1186/s41687-020-0181-7 32112189 PMC7048882

[B29] Schumaier A. P. Matar R. N. Ramalingam W. G. Archdeacon M. T. (2022). Patient-reported outcomes for fractures of the acetabulum: a comparison between patient-reported outcomes information system and traditional instruments. J. Am. Acad. Orthop. Surg. 30 (2), 71–78. 10.5435/JAAOS-D-20-01324 34543239

[B30] Strutzenberger G. Leutgeb L. Claußen L. Schwameder H. (2022). Gait on slopes: differences in temporo-spatial, kinematic and kinetic gait parameters between walking on a ramp and on a treadmill. Gait Posture 91, 73–78. 10.1016/j.gaitpost.2021.09.196 34653877

[B31] Tannast M. Najibi S. Matta J. M. (2012). Two to twenty-year survivorship of the hip in 810 patients with operatively treated acetabular fractures. J. Bone Jt. Surg. Am. 94 (17), 1559–1567. 10.2106/JBJS.K.00444 22992846

[B32] Verbeek D. O. van der List J. P. Tissue C. M. Helfet D. L. (2018). Long-term patient reported outcomes following acetabular fracture fixation. Injury 49 (6), 1131–1136. 10.1016/j.injury.2018.04.031 29729818

[B33] Warmerdam E. Huebner M. Stoll C. Lange A. I. Ganse B. (2025). Recovery of patient-reported outcome measures vs gait parameters obtained by instrumented insoles after tibial and malleolar fractures: prospective longitudinal observational study. JMIR Mhealth Uhealth 13, e71022. 10.2196/71022 40523278 PMC12209729

[B34] Wolff C. Steinheimer P. Warmerdam E. Dahmen T. Slusallek P. Schlinkmann C. (2023). Effects of age, body height, body weight, body mass index and handgrip strength on the trajectory of the plantar pressure stance-phase curve of the gait cycle. Front. Bioeng. Biotechnol. 11, 1110099. 10.3389/fbioe.2023.1110099 36873371 PMC9975497

