# Supplementary material for: Impaired patient-reported outcomes but preserved gait patterns 5–15 years after acetabular fracture compared with healthy controls
Source: Front Bioeng Biotechnol. 2026 Jan 15;14:1727785. doi: 10.3389/fbioe.2026.1727785 (PMC12852339; doi:10.3389/fbioe.2026.1727785)
Supplement: Supplementary file 1 [file DataSheet1.pdf]

Supplementary Material

1 Supplementary Tables

Patient	Sex [M/F]	BMI [kg/m^2]	Age at time of fracture [years]	Side (R/L)	Fracture type according to Judet and Letournel	Concomitant injury	Treatment (ORIF = open reduction and internal fixation)
1	M	24.95	35	L	Posterior wall	Femoral Head fracture	ORIF
2	M	37.82	26	L	Posterior wall	Contusion of shoulder, thorax and proximal fibula	Nonoperative
3	M	25.65	28	R	Both columns	Traumatic brain injury (mild), sternal fracture, pneumothorax	ORIF
4	M	28.25	18	R	Posterior wall	Traumatic brain injury (mild), thoracic trauma and laceration of the lower leg	ORIF
5	M	32.76	45	L	Anterior column	-	ORIF
6	F	21.46	19	L	Anterior column, posterior hemi-transverse	-	ORIF
7	M	33.06	49	L	Anterior column, posterior hemi-transverse	-	ORIF
8	M	28.71	43	R	Posterior wall	-	ORIF
9	F	24.95	57	L	Anterior column, posterior hemi-transverse	-	Nonoperative
10	F	24.98	52	L	Anterior column	-	Nonoperative
11	F	18.09	21	R	Anterior column, posterior hemi-transverse	-	Nonoperative
12	F	26.21	51	L	Posterior wall	Traumatic brain injury (mild), transverse process fracture L2-L5, knee contusion	Nonoperative
13	M	26.85	42	R	Anterior column, posterior hemi-transverse	-	ORIF
14	M	31.88	49	R	Both Columns	Radial head fracture	ORIF
15	F	22.40	31	L	Anterior Column	Nasal bone fracture	Nonoperative
16	M	27.72	34	L	Both columns	Traumatic brain injury (mild),<br>laceration at the back of the head	ORIF
17	M	26.59	40	L	Transverse	-	ORIF
18	M	25.88	66	R	Anterior column	-	Nonoperative
19	M	27.04	43	L	Anterior column, posterior hemi-transverse	-	Nonoperative
20	M	22.75	57	L	T-shaped	Basal phalanx fracture D5	ORIF
21	F	25.55	53	L	T-shaped	Pubic bone fracture (both sides)	ORIF
22	M	22.04	56	R	Posterior wall	-	ORIF
23	M	28.04	41	R	Anterior column, posterior hemi-transverse	Monteggia fracture	Nonoperative
24	M	25.63	58	R	Anterior column, posterior hemi-transverse	Urinary tract infection	ORIF
25	M	29.65	45	R	Posterior column	-	ORIF
26	M	27.65	39	L	Both columns	-	ORIF

Table i. Details on the patients that were included in this study.

Gait parameter	P-value
Vertical Impulse	0,892
Loading Rate	0,825
Loading Peak Force	0,57
Time To Loading Peak	0,838
Mid Support Force	0,773
Time To Mid Support	0,977
Push Off Peak Force	0,831
Time To Push Off Peak	0,819
Push Off Rate	0,573
Braking Impulse	0,473
Braking Peak Force	0,44
TimeTo Braking Peak	0,974
Propulsive Impulse	0,741
Propulsive Peak Force	0,977
Time To Propulsive Peak	0,71
Contact Duration	0,666
Step Duration	0,518
Double Support Duration	0,6
Single Support Duration	0,263
Stride Duration	0,809

Table ii. Gait parameters and P-values Comparison of gait parameters of patients and the healthy control subjects. No significant differences were found between the patients and the healthy control subjects in any of the tested gait parameters.
